# Shedding light on biology of bacterial cells

**DOI:** 10.1098/rstb.2015.0499

**Published:** 2016-11-05

**Authors:** Johannes P. Schneider, Marek Basler

**Affiliations:** Focal Area Infection Biology, Biozentrum, University of Basel, 4056 Basel, Switzerland

**Keywords:** imaging, live cells, bacteria

## Abstract

To understand basic principles of living organisms one has to know many different properties of all cellular components, their mutual interactions but also their amounts and spatial organization. Live-cell imaging is one possible approach to obtain such data. To get multiple snapshots of a cellular process, the imaging approach has to be gentle enough to not disrupt basic functions of the cell but also have high temporal and spatial resolution to detect and describe the changes. Light microscopy has become a method of choice and since its early development over 300 years ago revolutionized our understanding of living organisms. As most cellular components are indistinguishable from the rest of the cellular contents, the second revolution came from a discovery of specific labelling techniques, such as fusions to fluorescent proteins that allowed specific tracking of a component of interest. Currently, several different tags can be tracked independently and this allows us to simultaneously monitor the dynamics of several cellular components and from the correlation of their dynamics to infer their respective functions. It is, therefore, not surprising that live-cell fluorescence microscopy significantly advanced our understanding of basic cellular processes. Current cameras are fast enough to detect changes with millisecond time resolution and are sensitive enough to detect even a few photons per pixel. Together with constant improvement of properties of fluorescent tags, it is now possible to track single molecules in living cells over an extended period of time with a great temporal resolution. The parallel development of new illumination and detection techniques allowed breaking the diffraction barrier and thus further pushed the resolution limit of light microscopy. In this review, we would like to cover recent advances in live-cell imaging technology relevant to bacterial cells and provide a few examples of research that has been possible due to imaging.

This article is part of the themed issue ‘The new bacteriology’.

## 1. Imaging techniques

Because a typical bacterial cell is only 1 µm wide and up to 5 µm long, live-cell fluorescence imaging of bacteria has its specific challenges. Conventional wide-field epifluorescence microscopy is to date the most commonly used method to study subcellular features in bacteria. The lateral resolution of this technique is limited to approximately 200–250 nm by the diffraction barrier and the axial resolution is limited to approximately 500–700 nm. Laser scanning confocal microscopy (LSCM) can produce improved axial resolution and contrast in samples thicker than the focal plane (approx. 0.5 µm) owing to the focused laser beam and the pinhole in front of the detector that significantly reduces out-of-focus haze. However, considering the small size of bacteria, major disadvantages of LSCM are the scanning process, which limits temporal resolution, and the need for more excitation energy resulting in increased photobleaching. Therefore, LSCM is usually applied when three-dimensional (3D) sectioning of a sample is required to study interactions of pathogens with eukaryotic cells [1–3] or organization of bacterial biofilm structures [4,5]. The signal-to-noise ratio can be significantly improved in both LSCM and epifluorescence microscopy using deconvolution algorithms, although this requires a considerable computing power and may be time consuming [6–8]. Total internal reflection microscopy (TIRF) and highly inclined laminated optical sheet microscopy (HILO) exploit advantages of using different sample penetration depths. This enables single molecule tracking in or close to the membrane using TIRF [9,10] and visualization of cytoplasmic organized structures via HILO [11–13] in live bacteria.

One of the most important developments during the last two decades was clearly the advent of super-resolution imaging [14]. Breaking the diffraction barrier has enabled imaging of structures in eukaryotic as well as bacterial specimens with highest precision. Depending on the super-resolution approach being used, lateral resolution of single objects can be pushed to 45–62 nm in structured illumination microscopy (SIM) [15], to approximately 50 nm in stimulated emission depletion (STED) [14] and to approximately 20 nm in photoactivated localization microscopy (PALM) or stochastic optical reconstruction microscopy (STORM) [16,17]. All these techniques have certain advantages and disadvantages that need to be considered for experimental set-up, and live-cell imaging has been especially challenging for several reasons (see table 1 or a more detailed description in Yao & Carballido-Lopez [18]). When super-resolution data are processed and analysed, it is very important to ensure that no artefacts are generated during the process by choosing adequate controls [19,20]. Arguably, the most popular approach for live-cell super-resolution imaging to date is SIM, because common fluorophores can be used. Moreover, SIM allows the generation of a reconstructed super-resolution image with acquisition times of 0.1 to a few seconds [15,21,22]. So far, STED imaging of live cells has been much more challenging due to the high energy of light that is required for the process of STED and the resulting phototoxicity and bleaching [15,23]. New approaches that use extremely stable fluorophores [24,25] or gated-STED (G-STED) [26,27] are now able to overcome this limitation. Although the application of localization-based techniques is currently limited in live-cell imaging by comparably long acquisition times, the use of small molecules and their relatively poor applicability to bacterial systems, as well as photon yield of fusion proteins such as mEos or PAmCherry, several improvements have been implemented in recent years to bypass these problems [28–30]. In addition, considerable progress has been made using different PALM modes in bacterial live-cell imaging [19,31–34]. For further reading, we recommend an excellent review by Yao & Carballido-Lopez highlighting diverse aspects of bacterial live-cell imaging techniques and applications [18].

**Table 1.** Comparison of imaging techniques. TIRF, total internal reflection fluorescence; HILO, highly inclined laminated optical sheet; STED, stimulated emission depletion; SIM, structured illumination microscopy; PALM, photoactivation localization microscopy; STORM, stochastic optical reconstruction microscopy. For references, see text.

	diffraction-limited				super-resolution		
	wide-field	confocal	TIRF	HILO	STED	SIM	PALM/STORM
lateral resolution *x*, *y* (nm)	200–250	180–250	200–250	200–250	∼50	∼50–100	∼10–20
axial resolution *z* (nm)	500–700	500–700	∼100	500–700	∼20–100	∼200–300	∼20–100
limitations	poor contrast	weaker intensity	surface only	uncertain depth	high phototoxicity	data processing, image reconstruction artefacts	slow, data processing
advantages	fast, sensitive	optical sectioning	fast, sensitive	high contrast	fast, no data processing	all probes	highest resolution

The combination of different cutting-edge microscopy approaches as well as complementation with other technologies now presents opportunities to study cells in unprecedented detail. Recently developed techniques include correlated cryo-PALM-CET (cryo-electron tomography) [35], cryogenic super-resolution correlative light and electron microscopy (csCLEM) [36], correlative multicolour 3D SIM and STORM [37], instant live-cell super-resolution imaging via nanoinjection-based labelling [38], scanning electrochemical microscopy combined with micro-3D printing [39] and correlative live-cell and super-resolution microscopy [40]. However, these recent technological advances have so far been applied primarily in eukaryotic cells and adaptation for bacterial cell imaging could be challenging, because of the smaller size and volume and the specific procedures (e.g. washing of excess dye) needed to successfully generate high-quality images.

## 2. Tags

Green fluorescent protein (GFP) has been used as genetically encoded fluorescent reporter for gene expression and protein localization studies since the first description in 1994 [41] and variants developed now cover the whole spectrum of visible light—from the 355 nm excitable Sirius [42] to 702 nm excitable near-infrared iRFP720 [43]. Importantly, one should consider that the application of specific tags and the desired readout, namely fluorescence, might be disturbed by autofluorescence of inherent compounds such as flavin or NADH/NADPH. Depending on the nature of the compounds or their presence in different species, background fluorescence could be generated at different wavelengths [44]. Usually, this is circumvented by application of tailored filter sets in modern microscopes that allow the discrimination of fluorescence of a particular tag from the cellular autofluorescence. One of the major drawbacks of commonly used fluorescent proteins (FPs) is their tendency to oligomerize when they are fused to a protein of interest. This may result in the formation of foci-like structures, usually at the cell pole, or alter the dynamics of the protein of interest. This problem is especially pronounced for proteins that form oligomeric structures [12]. This most probably happens due to avidity effects where the FP acts as scaffold promoting unspecific aggregation [12]. To circumvent this problem, monomeric versions of many FPs were generated by introducing charged residues (e.g. the A206 K mutation in GFP variants) [45]. Oxygen dependence of many commonly used fluorophores has been an issue preventing experiments in anaerobic conditions [46] and long maturation time as well as low photostability has hindered observation of processes in real time [47]. The size of GFP and its derivatives (approx. 25–30 kDa) presents another downside, because it might interfere with protein folding, function or localization of fusion proteins. Furthermore, compared with organic fluorophores, FP quantum yield (*Φ*) is much lower, which limits their use in several applications, for example, in super-resolution microscopy. Such demanding approaches thus are pushing the development of alternatives.

In this section, we discuss selected probes, focusing especially on recent developments and applications for bacterial live-cell imaging (see also table 2).

**Table 2.** Recommended labels for different techniques. ++, very good; +, good; (+), possible, but not demonstrated yet; —, poor/not applicable; FP, fluorescent protein; pa-FP, photoactivatable FP; ps-FP, photoswitchable FP; pc-FP photoconvertible FP; TC-tag, tetracystein-tag; UAA, unnatural amino acid incorporation; FbFPs, flavin binding fluorescent proteins; FA-tag, fluorescence-activating tag. For references see text.

	diffraction limited	super-resolution		
		STED	SIM	PALM/STORM
‘conventional’ FPs	++	++^b^	++^b^	—
pa-FPs/ps-FPs/pc-FPs	+	+	+^c^	++
SNAP-/ CLIP-/HALO-tag^a^	++	++	++	++
TC-tag	++	(+)	(+)	+
UAA^a^	++	++	++	++
FbFPs	+	—	(+)	+
FA-tag	++	(+)	(+)	(+)

^a^Only in combination with small organic dyes.

^b^High fluorophore stability and brightness required.

^c^Only with pc-FPs.

### (a) Recent advances in natural cofactor-based fluorescent proteins

Near-infrared FPs were engineered from bacteriophytochromes that incorporate biliverdin, allowing deep-tissue imaging in living organisms due to long excitation/emission wavelengths of light with very low absorption [48,49]. Almost all variants of near-infrared FPs, however, are dimeric and the first monomeric variants described were later found to be dimeric at higher concentrations [50]. Only recently has a true monomeric version (mIFP) been developed and used for *in vivo* imaging in eukaryotic cells by Yu *et al.* [50].

A very interesting class of FPs that are now becoming more and more popular is flavin-based fluorescent proteins (FbFPs). These proteins harbour LOV (light, oxygen or voltage sensing) domains that bind flavin mononucleotide (FMN) thus rendering blue-light absorbing FPs. Overall, they are small, pH and thermostable FPs that also perform well under anaerobic conditions. One FbFP variant known as miniSOG can additionally generate reactive oxygen species, making it suitable for applications like correlated light and electron microscopy [51,52]. One of their major advantages might be, however, that FbFPs are particularly small (12–16 kDa) compared with GFP and its variants (25–30 kDa). Recently developed monomeric versions of these proteins exhibit quantum yields between 0.2 (phiLOV2.1) [53] and 0.51 (CreiLOV) [54]. These proteins are promising candidates to circumvent the problem of steric hindrance of a larger GFP molecule. Interestingly, Losi *et al.* [55] demonstrated that the LOV domain-containing photoreceptor YtvA of *Bacillus subtilis* can be used for super-resolution microscopy (Fluorescence PhotoActivation Localization Microscopy or FPALM) in bacteria. Nevertheless, to date there are few bacterial *in vivo* studies using FbFPs thus more are needed to assess the full capacity of LOV-based reporters [54]. A detailed review on FbFPs can be found in Buckley *et al.* [56].

Another member of natural cofactor-based FPs is UnaG, a small (approx. 14 kDa) protein of the Japanese eel (unagi), which was found to bind bilirubin with high affinity and specificity [57]. This non-covalent binding of the cofactor induces fluorescence even under anaerobic conditions [57]. Even though bacteria do not produce bilirubin, it can be added to the medium as it can pass the membrane and bind to UnaG in the cytosol [57].

### (b) Chemical labelling of proteins or polypeptides

All previously mentioned examples of proteins exploit the interactions with natural occurring substrates. Interestingly, there is a variety of peptide or protein tags that are non-fluorescent on their own, but are able to specifically bind synthetic compounds. This class includes, for example, so-called fluorogen-activating proteins (FAPs) [58,59]. FAPs were initially restricted to cell surface labelling [58] but have been evolved to also target intracellular proteins of interest in recent years [60–62]. A targetable near-infrared photosensitizer (TAP) FAP that has been described by He *et al.* allows researchers to study protein inactivation, targeted-damage introduction and cellular ablation with unprecedented precision [63].

Tagging systems also belonging to this category are SNAP-tag [64], CLIP-tag [65] and HALO-tag [66]. SNAP-tag labelling is very popular in the eukaryotic field, partially due to the commercial availability of many different substrates that are often tailored for specific uses. One of the latest additions to the palette of SNAP-/CLIP-tag or HALO-tag fluorogens are near-infrared silicon-containing rhodamine (SiR) dyes that can be used in super-resolution applications [67]. Interestingly, in 2011, Sun *et al.* described the fast-labelling variant SNAP_f_, which allows labelling without or with very little washing [68]. Because most chemical labelling approaches require multiple washing steps in order to reduce fluorescence signal of unreacted probes, thus potentially limiting several applications, the use of this tag might be advantageous.

SNAP-tag labelling of bacterial fusion proteins for fluorescence microscopy has initially been performed with fixed *Escherichia coli* cells due to the high amount of dye that remains in the bacterial cells after staining and the corresponding excessive washing needed [12]. Imaging of SNAP-tag fusion proteins in live *E. coli* cells has been achieved using quenched SNAP-tag substrates [69]. However, labelling efficiency varied significantly and a comparably low signal-to-noise ratio was reported [69]. When these substrates were further analysed in eukaryotic cells, highly unspecific staining of cellular structures was observed [70]. Altogether, SNAP-tag labelling seems to be a very efficient and reliable system in eukaryotic cells, however, imaging of live bacterial cells expressing SNAP-tag labelled proteins still faces considerable challenges. Nevertheless, a few groups have achieved SNAP-tag labelling in living bacteria [31,71,72] and continuous development of fluorescent compounds as reported recently by Grimm *et al.* [73] might in fact increase the amount of possible applications and make this system more attractive for the prokaryotic field. Furthermore, specific HALO-tag labelling of cytoplasmic and periplasmic proteins in *E. coli* was quite recently achieved in a study by Ke *et al.* [74].

Considerable progress on a fluorescence-activating tag has been recently achieved by engineering variants of cellular retinoic acid binding protein II (CRABPII). CRABPII is a small protein (15.6 kDa) that belongs to the family of intracellular lipid-binding proteins and mutants have earlier been shown to form iminium-based pigment with retinal, thus rendering a colorimetric pH sensor [75]. Yapici *et al.* have further manipulated this system by generating CRABPII variants that covalently bind cell-permeable merocyanine aldehyde, which is non-fluorescent on its own [76]. This leads to the formation of an iminium and generates a permanent resonating cation, resulting in a red-shifted cyanine dye [76]. The CRABPII/chromophore complex is pH stable and has red-shifted emission (605–619 nm). In addition to that, it not only yields high quantum efficiency (up to 39%) but also exhibits slightly faster photobleaching when compared with mRFP [76]. In their study, Yapici *et al.* further applied this technique to stain live *E. coli* cells expressing a mutant CRABPII. Fluorescence appeared highly specific (minimal background) and very fast (less than 1 min), supporting *in vitro* assayed properties of the tag [76]. Altogether, this fluorescence-activating tag seems to be a very useful tool, harbouring different properties to commonly used FPs and thus making it an interesting new probe for imaging approaches in live cells.

Quite recently, a yellow fluorescence-activating and absorption shifting tag (Y-FAST) has been evolved from PYP-tag [77]. Y-FAST is an approximately 14 kDa, monomeric protein fusion tag that binds a non-toxic, cell-permeable fluorogen (HBR or HMBR). HBR and HMBR exhibit strong yellow fluorescence when bound to Y-FAST and excited with blue light. Moreover, these compounds can be obtained relatively easily by one-step chemical synthesis. A key advantage of Y-FAST is that binding of the substrate is instantaneous (milliseconds), highly specific and also fully reversible. Binding of H(M)BR to Y-FAST significantly increases quantum yield and also induces an absorption red shift. Owing to the remarkable properties and reaction dynamics it is applicable to various systems/organelles/cell types, allowing, for example, the following of protein synthesis in near real-time or enabling demanding *in vivo* multiplex imaging experiments. On top of that, the authors mention that Y-FAST should in principle behave as a blinking fluorophore and thus may be also used in various super-resolution approaches [78].

### (c) Bioorthogonal labelling

Site-specific labelling of proteins using click chemistry, incorporation of unnatural amino acids (UAA), deprotection reactions and ligand-directed chemistry is gaining increasing attention due to nearly unlimited possibilities to track molecules in living cells. To date, a number of these approaches have been successfully applied in live-cell imaging of eukaryotic and bacterial cells [79–85]. However, the field still faces several challenges regarding binding specificity, toxicity, synthesis or availability of compounds and cell permeability of reagents, which need to be addressed in the future [86].

One of the most promising bioorthogonal labelling systems is the tetracystein (TC) tag with its substrates FLAsH and ReAsH. FLAsH and ReAsH are membrane-permeable biarsenical compounds that are non-fluorescent when they are bound to ethane dithiol (EDT_2_). The FLAsH-/ReAsH-EDT_2_ compounds specifically bind to TC sequences that can be fused to a given protein of interest. Upon binding, the TC motif displaces bis-EDT and the tag becomes highly fluorescent, while unbound compounds stay non-fluorescent thus minimizing the need for excessive washing [87]. Another advantage of this technique is that possible stereochemical hindrance in fusion proteins can be avoided due to the small size of TC tags. TC sequences preferentially used are six amino acid (CCPGCC) [87] or 12 amino acid tags (HRWCCPGCCKTF and FLNCCPGCCMEP) [88]. Importantly, it is also possible that the compounds may bind to other thiol-rich proteins in the cell and thereby generate background signals, which in turn would require extended washing procedures. This can be easily evaluated using a non-labelled control. Moreover, fusion of cysteine-rich sequences to a given protein of interest might cause incorrect disulfide-bond formation and thus loss of protein integrity [89]. Besides that, FLAsH and ReAsH tags can be used in pulse chase experiments, chromophore-assisted light inactivation experiments (CALI), Förster resonance energy transfer (FRET), correlated light and electron microscopy (CLEM) and PALM [90–94].

TC-FLAsH/ReAsH has, for example, been used to visualize synthetic glycomodule peptides in plants [95], to follow secretion of Type 3 Secretion System effectors of *Shigella flexneri* into the host cell [96] and also to study lipid raft association of Ebola virus protein VP40, which is a crucial component of virus assembly and budding [97]. Moreover, a number of studies have applied this technique to localize TC-tagged proteins in live *E. coli* and *Caulobacter crescentus* cells using fluorescence microscopy [98–101].

Very comprehensive reviews highlighting the latest biorthogonal labelling strategies can be found in the references [86,102,103].

## 3. Fluorescence microscopy as a tool to answer biological questions

Live-cell fluorescence microscopy of bacteria has allowed researchers to understand many fundamental biological processes. Here, we highlight a few examples to illustrate the impact of recent progress in live-cell imaging. We also recommend the following detailed reviews: [104–107].

### (a) Cell shape

One of the most studied systems in bacteria is the cell shape-determining programme that leads to the formation of rod-shaped cells. Key components of this system are the actin homologous proteins Mbl/MreB/MreBH, which form the ‘bacterial cytoskeleton’. Observations based on fluorescence microscopy studies enabled researchers to develop a picture of how bacteria are able to generate and maintain the three-dimensional rod shape during growth and division. Early fluorescence microscopy studies found that Mbl formed dynamic helical filaments, ‘rotating’ inside bacterial cells [108]. From then on, many studies followed showing filamentous and rotating MreB/Mbl proteins and the first links to cell wall synthetic machinery were found. In 2011, however, the picture changed when three studies showed that MreB formed dynamic patches rather than relatively long filamentous structures, that the movement depended on peptidoglycan synthesis and that it was rather circumferential by using TIRF [9,10] and a similar approach [109]. By the end of 2012, Swulius & Jensen [110] showed that the filamentous helix observed with the YFP-MreB fusion in *E. coli* was indeed an artefact caused by the YFP tag. Using RFP fused to an internal loop of MreB and a combination of electron and light microscopy approaches, their data further supported the model of native MreB forming patches or short filaments [110]. However, application of cutting-edge super-resolution microscopy in *B. subtilis* and cryo-electron microscopy in *C. crescentus* in the following years by two groups provided strong evidence for the existence of filamentous MreB structures guiding the insertion of new cell wall material [111,112]. Interestingly, Treuner-Lange *et al.* recently elucidated a new role of the actin homolog MreB by studying focal adhesion-related gliding motility in *Myxococcus xanthus*. Using a fluorescence microscopy-based approach, the authors found that the cytoskeleton protein functions not only as scaffold in PG synthesis, but also independently acts as a scaffold that recruits components of the Agl-Glt gliding machinery like MglA, a Ras-like small G-protein [113]. Therefore, these results raise the exciting possibility that G-protein–cytoskeleton interactions are a universally conserved feature [113].

### (b) Secretion systems

Bacterial secretion systems evolved to deliver proteins from the bacterial cytosol, where they are synthesized, to the extracellular space or directly across the target cell membrane. These secretion systems have a diverse structure and mode of action [114]. In recent years, live-cell fluorescence microscopy has been used to understand the localization, assembly and dynamics of these systems.

#### (i) Type II

The type II secretion system secretes proteins from periplasm to the extracellular space through an outer-membrane secretin by a piston-like mechanism using a periplasmic filamentous pseudopilus [115]. Fluorescence imaging was used to localize two components of the inner membrane platform and revealed that in *Vibrio cholerae*, EpsC and EpsM form several discrete foci on the cell periphery [116]. This localization was dependent on the presence of several critical components of the T2SS and the number of foci correlated with level of expression and secretion activity of the T2SS. It was also noted that upon overexpression, these two components had a tendency to relocalize to the cell pole.

#### (ii) Type III

Here, we will note only a few interesting examples of studies that used fluorescence microscopy techniques to unravel important aspects of injectisome and flagellar functionality. Flagella are an example of the most sophisticated cellular machines in bacteria known to date. Microorganisms are able to rotate these long filaments in order to propel themselves through liquid environments. Flagella are formed via sequential self-assembly of the motor, hook and filament [117]. The motor complex is associated with a protein secretion apparatus that is energized by proton motive force and exports hook and filament components through the cell envelope [118]. Live-cell imaging was mainly used to understand the assembly and dynamics of these systems. For example, a combination of TIRF and FRAP showed that there are 22 copies of MotB protein in the flagellar motor of *E. coli* and that these components are dynamically exchanged with the pool of MotB in the membrane [119]. In 2011, Li & Sourjik [120] used a variety of fluorescence microscopy-based approaches to elucidate the step by step assembly of the flagellar motor components in *E. coli*, which led to a much more comprehensive view of this intricate machinery.

The flagellar secretion machinery is closely related to the syringe-like injectisome, a needle complex that is used by many Gram-negative pathogens to inject their effectors into eukaryotic host cells [121]. The underlying mechanism of protein export in both systems is termed type III secretion (T3S) [121,122]. Distribution around the bacterial cell and functional assembly of the *Yersinia* type III secretion injectisome was elucidated in two consecutive studies using fluorescence microscopy and biochemical approaches [123,124]. Here, Diepold *et al.* could demonstrate that two independently assembled complexes, consisting of (i) membrane rings that anchor the type III machinery in both bacterial membranes and the peptidoglycan, as well as (ii) the inner membrane complex and export apparatus get connected by the lipoprotein YscJ. This merger ultimately renders a functional injectisome [124]. Later, a combination of fluorescence microscopy and *in situ* cryo-electron tomography showed that under secretion-inducing conditions, injectisomes cluster within 100 nm and that the number of injectisomes per cluster increases [125]. Another study by Diepold *et al.* using PALM and FRAP further revealed the assembly of the cytosolic C-ring of the injectisome, showing that the C-ring is dynamic under secreting conditions but stable under non-secreting conditions [126]. Imaging was also used to estimate the rate of effector secretion by T3SS [96,127].

#### (iii) Type IV

The bacterial type IV secretion system (T4SSs) can translocate both DNA and proteins into bacterial or eukaryotic target cells [128]. Several components of T4SS were successfully tagged with FPs and subcellularly localized in live *Agrobacterium tumefaciens* cells [129,130]. GFP fusions to inner membrane structural component VirB8, VirD4 ATPase, as well as substrate proteins VirD2, VirE2 and VirF were expressed from native locus [129].

The combination of live-cell fluorescence microscopy and immunofluorescence microscopy showed that T4SS localize to the poles and also form helically arranged foci, colocalizing with MinD around the perimeter of the bacterial cell. These data supported a model where multiple T4SSs around the bacterial cell provide multiple sites for interaction with the host cell surface [129].

#### (iv) Type VI

The type six secretion system (T6SS) is one of the bacterial nanomachines that deliver proteins from the bacterial cytosol directly to an extracellular space. It resembles a contractile tail of a bacteriophage that is anchored to the bacterial envelope. Interestingly, the sheath-tube complex of this nanomachine was shown by cryo-electron microscopy to stretch across the whole bacterial cytosol of *V. cholerae*. This allowed monitoring sheath assembly and dynamics using live-cell fluorescence microscopy [131]. Because the whole sheath is composed of hundreds of copies of the same subunit, the overall brightness of labelled T6SS sheath is very high [131,132]. This enabled high-speed imaging of sheath assembly, contraction and disassembly. It was shown that the assembly as well as disassembly by the T6SS-specific ATPase, ClpV, takes tens of seconds [131,133]. The contraction was, however, shown to be too fast to time resolve as it takes less than 5 ms [131].

Since the initial imaging of sheath assembly and disassembly in *V. cholerae*, similar FP fusions were used to describe the dynamics of T6SS in many more organisms such as *E. coli*, *Serratia marcescens*, *Pseudomonas aeruginosa* and *Burkholderia* [134–137]. Imaging of T6SS components was also crucial for unravelling an intricate regulatory mechanism, which regulates subcellular localization of T6SS assembly in *P. aeruginosa* [136,138,139]. It was shown that *P. aeruginosa* cells are capable of responding to attacks from neighbouring cells, likely by sensing damage to their own membranes [136,138,140].

More recently, several components of the baseplate and membrane complex were successfully tagged and localized within bacterial cells [135,141,142]. This showed that only a limited amount of initiation spots are present in a cell and described the hierarchy of the assembly process. Live-cell imaging played a crucial role in establishing the role of the T6SS component TssA. The TssA protein was shown to interact with many components of the baseplate, but also the tube-sheath complex. Based on such data, it would be possible to conclude that TssA is a member of the baseplate complex. However, live-cell imaging showed that TssA is involved in initiation of sheath assembly and, importantly, that it is located at the end of a growing sheath [143].

### (c) Functional compartmentalization in bacteria

Traditionally, eukaryotic cells have been considered as evolutionarily complex cells because of their capacity to organize cellular processes in organelles like the nucleus or mitochondria, which makes them very efficient and robust. It was assumed that prokaryotes are much simpler organisms due to the apparent lack of such cellular compartments. However, over 60 years ago it was discovered that bacteria contain so-called micro-compartments (BMC) [144], which were later found to spatially separate metabolic processes in those cells [145,146]. In order to study the subcellular organization of bacterial cells, the application of fluorescence microscopy and electron microscopy techniques has been an invaluable tool for researchers. Today, it is known that several bacterial species are able to use different compartmentalization strategies in order to gain a selective advantage [147]. For example, it was shown that magnetosomes, the membranous organelles of magnetotactic bacteria, are spatially organized via cytoskeletal filaments [148]. In addition, one of the most studied phenomena in bacteria is the process of spore formation that effectively divides the bacterial cell into two physically separated compartments. Studying sporulation allowed researchers to gain detailed insight into basic yet very sophisticated and complex cellular processes such as morphogenesis and the organization of spatio-temporal gene expression [149]. In 2006, a study by Wagner *et al.* [150] indicated that another type of physical compartment exists in Gram-negative *C. crescentus*, separating cell body from stalk. Indeed, it was later shown that stalked *C. crescentus* cells are compartmentalized by formation of diffusion barrier complexes, which physically separate regions in the stalk to confer physiological advantages to the growing cell by minimizing the effective volume [151]. A different mechanism of spatio-temporal organization in bacterial cells is the generation of protein concentration gradients. To date, several gradient systems including the *Schizosaccharomyces pombe* kinase Pom1 and its target Cdr2 regulating cell size and homeostasis, the *C. crescentu*s division inhibitory protein MipZ and its target FtsZ, the *E. coli* ParA and cognate ParB–*parS* complex controlling plasmid segregation and the polar oscillation of Min system ensuring septum formation at midcell in *E. coli* have been characterized in more detail and recently reviewed [152–154]. Remarkably, the oscillating Min- and FtsZ/FtsA-systems of *E. coli* were found to form self-organized patterns on the cellular membrane during cell division in an energy-dependent manner [154].

The placement of the cell division machinery at midcell in combination with regulatory mechanisms such as nucleoid occlusion enables bacteria to efficiently coordinate replication and segregation of their chromosomes in a spatio-temporal manner [155]. Interestingly, Montero Llopis and colleagues were able to demonstrate that mRNA translation can also be spatially organized in bacteria that use the chromosome matrix as a template [156]. Fluorescence microscopy-based approaches have contributed significantly to gaining detailed insight into chromosome dynamics in recent years. Overall, the most commonly used model organisms to study these systems are *B. subtilis*, *C. crescentus* and *E. coli*. Some of the most recent advances contributing to a more detailed characterization of chromosome dynamics include two studies describing the importance of the spatio-temporal organization of the Min and nucleoid occlusion system in *B. subtilis* [157,158] and a study by Fisher *et al.* describing the 3D organization of the *E. coli* chromosome with high temporal resolution by using four-dimensional imaging [159]. Remarkably, knowledge about the bacterial nucleoid and division proteins was recently applied to assess the efficiency of new FP-fusion proteins with the focus to provide new guidelines for multicolour imaging in bacterial cells [160]. Furthermore, Ptacin *et al.* have shown that the scaffold protein PopZ of *C. crescentus* generates a 3D subcellular microdomain, which can influence the segregation of centromeres by modulating ParA ATPase activity [161]. More recently, another study in *C. crescentus* has elucidated a mechanism of how DNA double-strand breaks (DSBs) could be repaired during chromosome resegregation [162]. Here, the authors used an inducible restriction enzyme to introduce DSBs and then monitored key players of the segregation mechanism using time lapse microscopy. This enabled them to observe that DSB repair occurred independent of the proximity to the origin of replication and also during ongoing DNA replication, thereby showing that chromosomes exhibit very dynamic movements to promote repair and maintain integrity in bacteria [162]. Intriguingly, it was shown that after sudden chromosome loss, bacteria are still able to grow, divide and synthesize proteins for a considerable amount of time [163]. The authors of this study also mention the possibility that these so-called ‘DNA-less bacteria’ could function as a novel vaccination strategy [163]. Altogether, using fluorescence microscopy in many studies has established a very good understanding of these systems. To further refine the understanding of chromosome dynamics, however, researchers ultimately will have to turn to advanced super-resolution techniques [164].

Eukaryotic cells contain a variety of subcellular compartments to spatially separate diverse cellular processes. This also includes the cellular membrane, which contains so-called lipid rafts [165,166]. These microdomain platforms are constituted by certain lipids such as cholesterol and sphingolipids and contain a specific set of proteins, mainly related to signal transduction and protein trafficking. The integrity of lipid rafts is very important for the functionality of associated signalling networks and disruption of raft integrity can lead to severe physiological defects with various implications in health and disease [167–169]. Today, it is known that bacteria also harbour different types of lipid domains in their membrane to generate microenvironments for localization and activity of specific proteins [170]. This includes functional membrane microdomains (FMMs) described initially in *B. subtilis*, which are constituted by hydrophobic lipids, contain flotillin-like proteins and thus are postulated to be the bacterial equivalent to eukaryotic lipid rafts [171]. Recently, fluorescence and super-resolution microscopy-based approaches were used to demonstrate that bacteria organize a heterogeneous population of FMMs in the membrane via differential flotillin scaffold localization and thus are able to spatially and temporally separate the organization of different signal transduction networks [172]. Intriguingly, it has been demonstrated that in addition to genetic regulation, oxidative phosphorylation (OXPHOS) complexes of *E. coli* are spatially organized in the cellular membrane via different mechanisms: (i) by the formation of supercomplexes [173], (ii) polar segregation [174], and (iii) localization in membrane microdomains [175]. As flotillins, the marker proteins of FMMs, have been shown to act as molecular protein scaffolds [172,176] and also co-occur and directly interact with OXPHOS complexes [172,177], it seems likely that these complexes might be organized in FMMs via flotillin activity [178].

### (d) Outlook

Altogether, the few examples mentioned in this review illustrate why fluorescence microscopy is one of the most important tools to explore biological processes in living cells. In addition, a number of super-resolution imaging systems are now commercially available, facilitating access to the super-resolution field especially for researchers without previous expert knowledge. Application of point-based super-resolution techniques is now reaching up to 20 nm resolution in live-cell imaging, while SIM offers an advantage in acquisition speed. Further development of these techniques, as well as combination with other cutting-edge approaches, such as cryo-electron tomography, will have a great impact on studying the intricate biology of bacterial cells. Super-resolution systems and corresponding software have been subject to tremendous improvements during the last few years. However, the main limitation now lies in the availability of tags that are suitable for demanding super-resolution approaches and allow the study of proteins of interest in live bacterial cells. Increased efforts in the development of smaller, more stable fluorophores, fluorescence-activating tags or direct chemical labelling of amino acids will surely be key to tackling these problems in the near future.
